# Outcomes of the Use of Fully Covered Esophageal Self-Expandable Stent in the Management of Colorectal Anastomotic Strictures and Leaks

**DOI:** 10.1155/2014/187541

**Published:** 2014-12-18

**Authors:** Chad J. Cooper, Angel Morales, Mohamed O. Othman

**Affiliations:** ^1^Department of Internal Medicine, Texas Tech University Health Sciences Center of El Paso, 4800 Alberta Avenue, El Paso, TX 79905, USA; ^2^Division of Gastroenterology, Texas Tech University Health Sciences Center of El Paso, 4800 Alberta Avenue, El Paso, TX 79905, USA; ^3^Department of General Surgery, Texas Tech University Health Sciences Center of El Paso, TX 79905, USA

## Abstract

*Introduction.* Colorectal anastomotic leak or stricture is a dreaded complication leading to significant morbidity and mortality. The novel use of self-expandable metal stents (SEMS) in the management of postoperative colorectal anastomotic leaks or strictures can avoid surgical reintervention. *Methods.* Retrospective study with particular attention to the indications, operative or postoperative complications, and clinical outcomes of SEMS placement for patients with either a colorectal anastomotic stricture or leak. *Results.* Eight patients had SEMS (WallFlex stent) for the management of postoperative colorectal anastomotic leak or stricture. Five had a colorectal anastomotic stricture and 3 had a colorectal anastomotic leak. Complete resolution of the anastomotic stricture or leak was achieved in all patients. Three had recurrence of the anastomotic stricture on 3-month flexible sigmoidoscopy follow-up after the initial stent was removed. Two of these patients had a stricture that was technically too difficult to place another stent. Stent migration was noted in 2 patients, one at day 3 and the other at day 14 after stent placement that required a larger 23 mm stent to be placed. *Conclusions.* The use of SEMS in the management of colorectal anastomotic leaks or strictures is feasible and is associated with high technical and clinical success rate.

## 1. Introduction

Anastomotic adverse events after partial colectomy include bleeding, leaks, fistulas, or strictures. Anastomotic leaks or strictures are a dreaded adverse event of colorectal surgery that can lead to a prolonged hospital stay and significant morbidity and mortality. An anastomotic leak is defined as a defect of the intestinal wall at the anastomotic site leading to a communication between the intra/extraluminal compartments [1]. The risk varies with the site of the anastomosis with those placed less than 5 centimeters (cm) from the anal verge being particularly vulnerable [2]. The incidence of an anastomotic leak depends on the location of the anastomosis with the highest incidence of 10–20% occurring with colorectal anastomosis [3]. The majority of patients suspected of having an anastomotic leak based on clinical assessment will undergo imaging studies to confirm the diagnosis. The usual approach for management of postoperative anastomotic leaks includes intravenous antibiotics, bowel rest, percutaneous drainage, parenteral nutrition, and surgical diversion [4]. The patient may require reoperation that consists of taking down the anastomosis and creation of an end colostomy. The distal portion of the bowel can be closed and left within the abdominal cavity or exteriorized as a mucous fistula. Postoperative patients with a colorectal anastomotic leak have a mortality rate of 25–35% [5].

The development of anastomotic strictures following colorectal surgery is a frequent problem, occurring in up to 30% of cases [6]. Anastomotic strictures eventually become symptomatic by presenting with signs of partial or complete intestinal obstruction. An anastomotic stricture is defined by the inability to pass a 12-millimeter (mm) diameter sigmoidoscope through the anastomosis [7].

The use of covered self-expandable esophageal metal stents (SEMS) has been described in the management of benign leaks, perforations, and fistulae of the esophagus, stomach, postsurgical anastomoses of the upper gastrointestinal (GI) tract, and the biliary tree [8]. Currently, CSEMS have been approved in the United States for use in the esophagus and bile ducts only. SEMS have been used for the treatment of malignant biliary obstruction or in malignant esophageal strictures [9]. The use of SEMS elsewhere in the GI tract and for other purposes warrants further investigation especially in the management of postoperative colorectal anastomotic leaks or strictures. In this case series, we present our experience with the temporary placement of covered esophageal SEMS in the nonoperative management of benign postoperative colorectal anastomotic leaks or strictures.

## 2. Specific Aims

The specific aim of this study was to evaluate the technical and clinical outcomes of fully covered SEMS placement for postoperative benign anastomotic leak or strictures that took place at a single endoscopic unit of tertiary university based hospital. This is a novel approach to the management of these anastomotic adverse events with limited existing literature on the topic.

## 3. Materials and Methods

A retrospective medical chart review was performed on all patients that had a covered self-expandable esophageal stent (CSEMS) placed for either a colorectal anastomotic stricture or leak at the Texas Tech University Health Sciences Center of El Paso Affiliated Hospital, University Medical Center of El Paso. This study was approved by the institutional review board (IRB) of Texas Tech University Health Sciences Center of El Paso, Texas, with the IRB number of E14044. Strict confidentiality and patient's privacy protection was maintained throughout the entire data collection process. At the time of the endoscopic procedure, the details of the procedure were explained to the patient and an informed consent was signed. However at the time of this study the procedure had already been performed. All patients were NPO after midnight and received 2 fleet enemas one day prior to the procedure. Depending on the postoperative clinical status of the patient, a liquid diet was given and advanced as tolerated.

### 3.1. Patient Population

Patients who underwent CSEMS for treatment of colorectal postoperative benign anastomotic leak or stricture from the period of January 1, 2011, through March 15, 2014, were included. The data collected pertained to patient demographics, type of postoperative anastomotic adverse events, characteristics of the stent placed, operative or postoperative adverse events, and clinical and technical outcomes.

#### 3.1.1. Inclusion Criteria

1. Males and females of age 18–70 years old.
2. Abdominal surgery for colonic adenocarcinoma, diverticulosis, or diverticulitis with the creation of an end to end anastomosis.
3. Diagnosis of a colorectal anastomotic stricture or leak based upon clinical manifestations and barium enema.

#### 3.1.2. Exclusion Criteria

1. A diagnosis of an anastomotic stricture or leak at a location other than the colorectal area.
2. Previous endoscopic dilation of colorectal anastomotic stricture.

### 3.2. Procedure Details

Self-expandable metal stent (WallFlex Fully Covered Esophageal Stents, Boston Scientific, Natick, Mass.) was used in this case series. These stents were not dedicated (esophageal stent) with a slight different radial force compared with colonic stent. A barium enema was performed within 1 week before and after stent placement. We did not use the fluoroscopy because we were inserting the endoscope and we placed stents under endoscopic visualization. The CO_2_ insufflator was used for all cases. An 18 mm stent diameter was used initially for stricture cases and 21 mm stent diameter was used for leak cases. In case of stent migration, 23 mm stent diameter was used for repeat stent placement. The choice of the stent length was dependent on stricture length.

**Figure 1:**
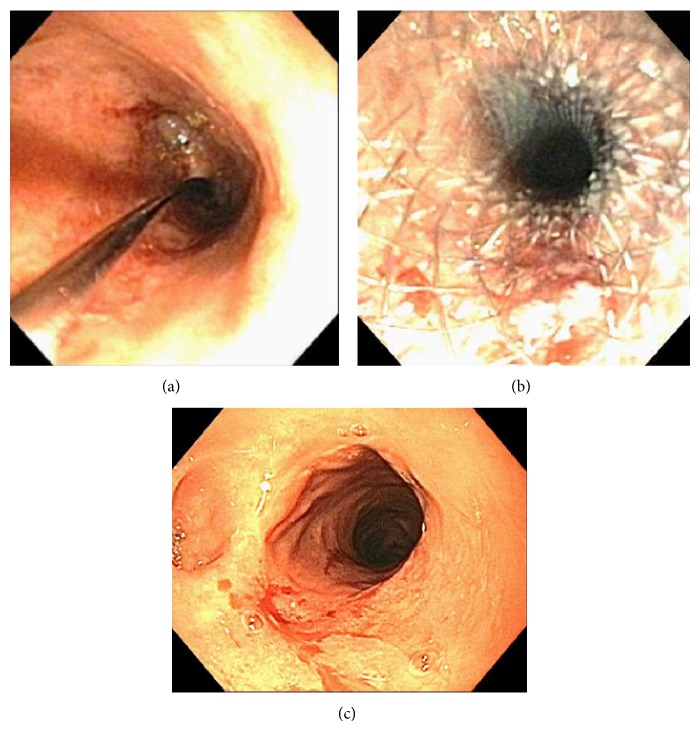
(a) Savory guidewire advanced through anastomotic stricture. (b) CSEMS deployment across the stricture. (c) Stricture resolution after stent removal.

A savory guidewire was placed endoscopically across the stricture of the leak (Figure 1(a)). The sigmoidoscope was then withdrawn and exchanged over the guidewire. The fully covered SEMS was then advanced over the guidewire and deployed under endoscopic visualization by advancing ultrathin endoscope (Olympus America Inc., Center Valley, PA) alongside the stent just below the stricture (Figure 1(b)). The stent was left at the anastomotic site for 40 to 50 days.

Endoscopic stent removal was performed with a rat tooth forceps (US Endoscopy, Mentor, OH) by grasping the distal end of the stent. Once the stent was removed a flexible sigmoidoscopy was performed to confirm healing of the leak or resolution of the stricture (Figure 1(c)). A final barium enema was performed within 3 months from stent removal to evaluate the status of the anastomotic defect before flexible sigmoidoscopy. A follow-up flexible sigmoidoscopy was performed between 3 to 4 months to evaluate recurrence of the defect. All flexible sigmoidoscopy procedures were performed under monitored anesthesia care (MAC) sedation.

## 4. Endpoints

Primary endpoints of the study were the clinical success that was quantified by the improvement in their quality of life, efficient bowel transit, and avoidance of surgical intervention. Each patient was followed up while hospitalized to evaluate their clinical status and postoperative pain. Further clinical and technical success was assessed as the resolution of the anastomotic leak or resolution of the anastomotic stricture as confirmed by flexible sigmoidoscopy at the time of stent removal and follow-up endoscopic and barium enema evaluation 4 to 6 weeks afterwards. Other primary outcomes included the technical success of stent deployment and removal. Technical success was considered to be a success if the stent deployed and was placed at the appropriate location of the anastomotic adverse event. Technical success at the time of removal was considered to be successful if the whole stent was removed without adverse events such as perforation. Secondary endpoints included complications such as stent migration, excessive bleeding, bowel perforation, or anorectal pain. Both immediate and delayed (>30 days after the procedure) adverse events such as stent migration were noted.

### 4.1. Statistical Analysis

All results were expressed as mean or percentage. Descriptive statistics such as means and percentages were used for continuous and categorical data, respectively.

## 5. Results

A total of 8 patients (5 males and 3 females) underwent flexible sigmoidoscopic placement of a fully CSEMS for management of a postoperative colorectal anastomotic leak or stricture. The mean age of the patients was 55.8 years.  Table 1 provides overall the patient demographics, anastomotic characteristics, stent characteristics, adverse events, and technical and clinical outcomes. The primary disease process included 5 patients with colorectal adenocarcinoma, 2 had diverticulitis, and 1 had diverticulosis. All 8 patients had a colorectal anastomosis. Five patients had a colorectal anastomotic stricture and 3 had a colorectal anastomotic leak. The SEMS were left in place for a mean of 45 (40–50) days. The distance from the distal flange of the stent to the anal verge ranged from 15 to 20 cm.

Clinical and technical success was initially achieved in all 8 cases (100%). However, two patients had external stent migration, one on postoperative day 3 and the other on day 14 after initial stent placement. Both of these patients had placement of a larger stent (23 mm stent diameter) and on follow-up complete resolution of the stricture was noted. Complete resolution of the anastomotic stricture or leak was achieved in all 8 cases (100%). Recurrence of the stricture after stent removal was not noted in any of these five patients. However, all 5 patients had a follow-up flexible sigmoidoscopy 3 months after stent removal. Three of the 5 patients had a recurrence of the anastomotic stricture that required placement of a CSEMS. Unfortunately, the anastomotic stricture was so severe in 2 of these patients in whom stent placement was not technically feasible. In the long term, 2 of the 5 (40%) patients with recurrence of the anastomotic stricture eventually failed endoscopic placement of another stent. These two patients eventually required an exploratory laparotomy with colorectal anastomosis revision approximately 6 months later. Therefore, in the anastomotic stricture subgroup, 60% of patients had resolution of the stricture. The other patient with recurrence of the stricture had a 23 mm × 15.5 cm stent placed with resolution of the stricture noted upon removal of the stent and also on follow-up flexible sigmoidoscopy 3 months afterwards. Anastomotic leak completely resolved in the three patients in our series. Two patients had mild rectal pain after the initial stent placement which was treated with oral analgesic. Endoscopic stent removal was successful in all cases.

## 6. Discussion

Covered self-expanding esophageal metal stents (CSEMS) are commonly used and proven successful for the temporary decompression of a malignant obstruction or as palliative therapy when surgery is not warranted [10–12]. CSEMS are flexible stents that allow some bowel wall movement that helps the stent conform to the bends in the bowel. Stent placement can allow for luminal contents to be diverted from the defect site and facilitate natural closure and resolution of the defect, thus preventing extraluminal fluid collection and infection. The use of SEMS may be associated with a longer lasting dilatation and a lower rate of recurrence in cases of anastomotic stricture or leak [13]. Furthermore, this minimally invasive technique can prevent the need for invasive procedures. There has been limited experience reported in the current literature regarding the use of this technique for colorectal anastomotic strictures, leaks, or fistulas. The reported adverse events of stent insertion include mucosal overgrowth, stent migration, and obstruction.

Currie et al. performed a systematic review on 122 patients that had a self-expandable stent in the management of benign colorectal obstruction [14]. The predominant etiology was diverticulitis (54%) followed by anastomotic stricture (33%). Technical success was achieved in 94% and clinical success in 87% of patients. Overall, the perforation rate was 12% and the reobstruction rate was 14%. Migration of the stent occurred in 20% of cases. The most common reasons for clinical failure were perforation in 10 patients, persisting obstruction in the presence of a technically successful stent in 8 patients, and excessive anal pain after insertion of the stent in 3 patients [14]. Their analysis revealed that diverticulitis had a greater risk of developing complications during stenting, possibly due to the underlying inflammation and scarring [14]. It has been suggested that there is an increased risk of migration with self-expandable metal stents due to the use of covered and uncovered metal prostheses, which lack the required flexibility, in fibrotic and noncompliant benign strictures [14].

Vanbiervliet et al. reported on 43 patients that had fully covered self-expanding metal stents for benign colonic strictures [15]. The most common etiology was anastomotic strictures in 40 (93%) patients. The stents were placed and removed approximately 4–* *6 weeks later. Clinical success was achieved in 35 (81* *%) patients. Migration of the stent was observed in 27 (63* *%) patients. The median duration of stenting was 21 days. Their analysis showed that stents >20* * mm in diameter migrated less often. Recurrence of stricture along with obstructive symptoms was observed in 23 (53* *%) patients. This study concluded that fully covered self-expandable metal stents for treatment of symptomatic benign colonic strictures are safe and effective, despite a high rate of spontaneous migration [15].

Abbas reported on 2 cases of an anastomotic stricture causing bowel obstruction in postoperative patients which were treated successfully with CSEMS placement [16]. Kim et al. reported a technical success of 100% and clinical success of 80% for the use of CSEMS for anastomotic leaks [6]. There was one of the five patients that had clinical failure but only had the stent placed for 20 days. This may not have been enough time to allow the anastomotic leak to completely heal. Sixty percent of their cases had postoperative adverse events of rectal pain and 40% had migration of the stent. Song et al. reported that rectal pain occurs in >60% of cases if the placement of the stent is within 5 cm of the anal verge [17].

The main limitation of this study is that it is a retrospective design with a small number of patients in this series. We therefore recommend further investigation of this minimally invasive technique in a large prospective controlled trial, given the potential benefits of this procedure. The recommended diameter for colonic stenting is 24 mm since it was determined as a cutoff regarding the migration risk. Nevertheless, our migration rate is lower than that observed in other studies; this could potentially be related to the severity of the stricture at the time of stent placement. Our study contributes to the available data on the use of esophageal CSEMS for management of postoperative colorectal leaks or strictures. Long term follow-up is required to evaluate the effectiveness and outcome of the procedure. We suggest a prospective comparative study (CSEMS versus dilation) to clearly establish the role of stenting in the treatment of the stricture. Furthermore, the use of CSEMS as a minimally invasive option and the avoidance of surgical intervention are very attractive in the treatment of anastomotic strictures or leaks. In conclusion, we recommend the use of CSEMS in the management of colorectal anastomotic leaks or strictures is feasible and is associated with high technical and clinical success.

## Figures and Tables

**Table 1:** Patient demographics, anastomotic/stent characteristics, adverse events, and clinical outcomes.

Patient	Age/sex	Primary disease process	End to end anastomosis location	Anastomotic stricture or leak	Stent type^*^	Stent size^#^	Days stent left in place	Procedure or postoperative adverse events	Technical success	Clinical success	Follow-up flexible sigmoidoscopy
1	62/M	Sigmoid/rectal CA	Colorectal	Leak	FC WallFlex	23 mm × 10.5 cm	50	No	Yes	Yes	4 months

2	63/F	Sigmoid CA	Colorectal	Leak	FC WallFlex	23 mm × 10.5 cm	50	No	Yes	Yes	4 months

3	64/F	Diverticulosis in sigmoid colon	Colorectal	Leak	FC WallFlex	23 mm × 10.5 cm	45	No	Yes	Yes	4 months

4	33/M	Sigmoid CA	Colorectal	Stricture	FC WallFlex	18 mm × 15.3 cm	40	Stent migrated externally (3 d); 23 mm × 12.5 cm placed	Yes	Yes	3 months

5	57/M	Rectal CA	Colorectal + fistula	Stricture	FC WallFlex	18 mm × 15.3 cm	40	No	Yes	Yes; but later required anastomotic revision	3 months; recurrence of stricture; unable to place another stent

6	58/F	Rectal CA	Colorectal	Stricture	FC WallFlex	18 mm × 15.5 cm	40	No	Yes	Yes	3 months; recurrence of stricture; 23 mm × 15.5 cm stent placed and stricture resolved

7	47/M	Diverticulitis in sigmoid colon	Colorectal	Stricture	FC WallFlex	18 mm × 15.3 cm	50	Stent migrated externally (14 d); 23 mm × 15.5 cm placed	Yes	Yes; but later required anastomotic revision	3 months; recurrence of stricture; unable to place another stent

8	63/M	Diverticulitis in sigmoid colon	Colorectal	Stricture	FC WallFlex	18 mm × 15.5 cm	50	No	Yes	Yes	3 months

^*^Esophageal stent (FC: fully covered).

^#^Shaft diameter (mm) × length (cm).

## Abbreviations

CSEMS: Covered self-expandable esophageal metal stents
MAC: Monitored anesthesia care
IRB: Institutional review board.
